# Characterizing Retail Food Environments in Peri-Urban Pakistan during the COVID-19 Pandemic

**DOI:** 10.3390/ijerph19148614

**Published:** 2022-07-15

**Authors:** Bianca Carducci, Yaqub Wasan, Agha Shakeel, Amjad Hussain, Jo-Anna B. Baxter, Arjumand Rizvi, Sajid B. Soofi, Zulfiqar A. Bhutta

**Affiliations:** 1 Centre for Global Child Health, Hospital for Sick Children, Toronto, ON M5G 0A4, Canada; joanna.baxter@mail.utoronto.ca; 2 Department of Nutritional Sciences, University of Toronto, Toronto, ON M5S 1A8, Canada; 3 Centre of Excellence in Women and Child Health, Aga Khan University, Karachi 74800, Pakistan; yaqub.wasan@aku.edu (Y.W.); aghashakeel@gmail.com (A.S.); amjad.hussain@aku.edu (A.H.); arjumand.rizvi@aku.edu (A.R.); sajid.soofi@aku.edu (S.B.S.); 4 Dalla Lana School of Public Health, University of Toronto, Toronto, ON M5T 3M7, Canada

**Keywords:** food environments, nutrition transition, South Asia, COVID-19

## Abstract

(1) Background: To date, there are limited data in low- and middle-income countries (LMICs) that collect, monitor, and evaluate food environments in standardized ways. The development of a pilot survey tool, tailored to LMICs and focused on retail food environments, is necessary for improving public health nutrition. (2) Methods: A novel survey tool was developed and piloted in a sample of village retail food environments (*n* = 224) in Matiari, Pakistan between October 2020 to April 2021. Villages were randomly selected, and food outlets were surveyed within a 500-m radius from each village center. Descriptive statistics (counts and percentages) were used to describe the characteristics of food outlets and the availability of food. To test whether there was a difference in characteristics or in the mean of number of healthy, unhealthy, and total food items available by village size, a χ^2^ test or one-way ANOVA was conducted, respectively. (3) Results: In total, 1484 food outlets were surveyed for food accessibility, availability, and promotion across small (*n* = 54), medium (*n* = 112), and large villages (*n* = 58). In small and medium-sized villages, mobile food vendors were the predominant food outlet type (47.8% and 45.1%, respectively), whereas in large villages, corner stores (36%) were more prominent. The mean number of total food items (*p* < 0.006) and unhealthy food items (*p* < 0.001) available in food outlets differed by village size. The proportion of food outlets with available fruits, meat and poultry, water, and sugar-sweetened beverages also differed by village size (*p* < 0.001). (4) Conclusions: This study informs the global evidence gap in the current understanding of food environments in various ethnically diverse and dynamic LMICs, and the developed methodology will be useful to other LMICs for measuring and monitoring the food environment, especially among vulnerable population groups. This work complements current national and provincial survey efforts in Pakistan.

## 1. Introduction

Diets and nutritional status have undergone a sequence of profound transformations over the past century, or namely nutrition transition [1]. Dynamic shifts in societies from agrarian to industrial food systems have been largely due to economic development, globalization, and urbanization [2,3] and have contributed to the rising rate of multiple forms of malnutrition or “Double Burden of Malnutrition” (DBM) [4]. In fact, the 2017 Global Burden of Disease study attributes 11 million deaths and 255 million disability-adjusted life years to suboptimal diets [5]. In South Asia, the DBM has rapidly grown over recent decades [2,3], and is expected to increase due to the COVID-19 pandemic [6]. Reduced diversity and increased homogenization of diets, as well as dramatic increases in the proportion of dietary energy from easily accessible and affordable foods high in fats, salt, and sugars have been well documented in the region [7].

According to the Sustainable Development Goals (SDG) Report 2021, progress is stalling in Pakistan on SDG 2, Zero Hunger, while SDG 3, Good Health and Well-being is moderately improving but insufficient to meet goals [8]. This is also reflective in the 2018 National Nutrition Survey whereby multiple forms of malnutrition are rampant among women, children, and adolescents in Pakistan [9]. Like other South Asian countries, Pakistan is experiencing a rapid increase in the DBM due to the size and aging of the population and a high degree of urbanization and lifestyle changes in favor of increased energy consumption and reduced physical activity [10]. In 2020, the population faced multiple shocks including high food prices, locust outbreaks, and heavy monsoon rains, which were exacerbated by the impacts of the COVID-19 pandemic [11]. To begin addressing the DBM, understanding food entry points is critical. 

The concept of the food environment describes “the collective physical, economic, policy, and sociocultural surroundings, opportunities, and conditions that influence people’s food and beverage choices and nutritional status” [12,13], which can be personal or external in nature and are reciprocal [14]. Standardized metrics and survey tools have been used extensively in high-income countries (HICs), such as the United States and Canada, to assess the food environments in neighbourhoods and near schools [15,16,17]. One of the most widely used tools is the Nutrition Environment Measures Survey (NEMS), [18,19], which has been adapted for use in several countries, including China [20]. Although the NEMS tools capture the availability and price of various (mostly healthy) foods, they do not capture nuances observed in informal retail settings and nutrition transition. Further, a comprehensive set of metrics (both objective and subjective) to measure external food environments have yet to be agreed upon [21,22]. Food environments in low- and middle-income countries (LMICs) are dominated by the informal sector (i.e., unregistered, mobile food vendors), as well as midstream micro-, small-, and medium-sized enterprises (MSMEs) [23]. Importantly, there have been very few studies conducted in South Asia that characterize external food environments at the market or retail level [24].

As such, the purpose of the current study is to: (1) design a novel survey tool that examines the retail food environment in terms of key food environment constructs in a peri-urban district of Pakistan and (2) examine key food environment constructs (i.e., accessibility, availability, and promotion) of food outlets by village size (small, medium, large).

## 2. Materials and Methods

### 2.1. Study Setting

The study was conducted in Matiari District which is part of the Province of Sindh in Pakistan. The population of Matiari was estimated to be around 770,000 in 2017 with approximately 76% being rural [25], representative of peri-urban Pakistan. Sindh has one of the highest rates of food insecurity, malnutrition, and poverty. Around 3.1 million people (26% of the rural population analyzed) were estimated to be facing high levels of acute food insecurity (Integrated Food Security Phase Classification Phase 3 or above) in the period of March to June 2021 [26].

### 2.2. Survey Tool Development

To determine appropriate survey items, we reviewed the literature on food environment assessments via Google Scholar and PubMed and supplemented with a “snowballing” method to search for other relevant information. Of the surveys identified, none were suitable to capture the nuances and cultural context of the Pakistani food environment, as they were intended for use in the United States. However, the NEMS instrument, which has been adapted for use in other LMICs [18,19,27,28], did provide a structure for conceptualizing a tailored, culturally relevant survey instrument comprised of various food outlet types (i.e., corner stores, restaurants, wet markets, street/mobile vendors, and fast-food) and constructs of the food environment: food accessibility, food availability, pricing/affordability, food promotion, and food policy (Table 1). 

For the purposes of this study, food accessibility, availability, and promotion were the primary food environment constructs [13,14]:

1. Accessibility measures included days and hours of operation, proximity to schools, and type of food outlet.
2. Availability referred to food availability and diversity of certain food and beverage products offered at different retailers (i.e., healthy food options vs. unhealthy food options).
3. Promotion referred to food advertising, marketing, and branding, and packaging directed at individuals through various mediums.

Based on local expert consultation, we aimed to incorporate a large variety of indicators that were practical and allowed for consistent and reliable measurement across contexts and over time. The initial survey tool was pretested in 90 food outlets by trained enumerators in February 2020. The pretest identified the removal of grains as a food group from the survey tool, since it is found in surplus, as well as missing details and the need for revision of wording and content. Due to constraints during the pandemic, data on economic access (i.e., food pricing) and food safety was not feasible and therefore removed. Furthermore, due to saturation, the decision to survey a maximum of 5 food outlets per type was agreed upon. The data collected during the pretest was not included in the analysis.

### 2.3. Sampling Strategy

**Figure 1:**
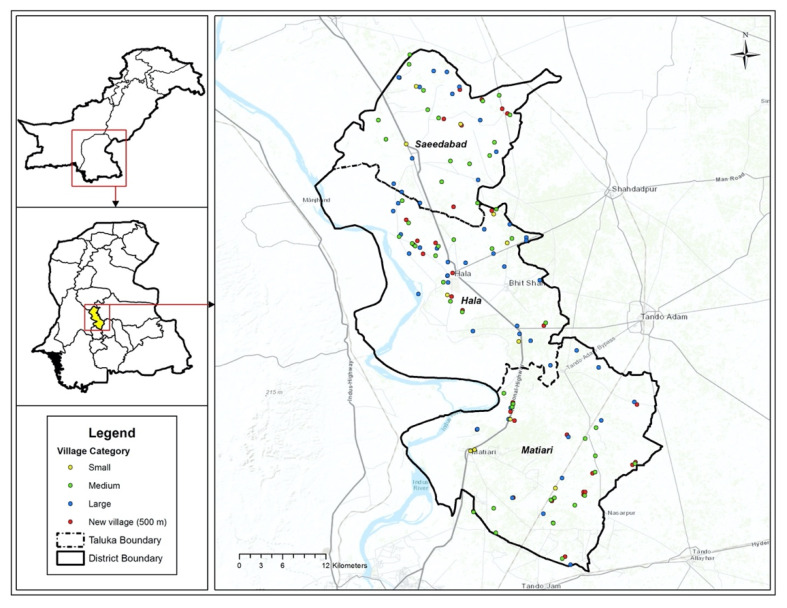
Map of Villages Surveyed in Matiari.

The prevalence of food insecurity was the primary outcome used for the analysis of precision in the sample size calculation. A two-sided 95% Confidence Interval (CI) for the one-proportion CI formula (simple asymptotic) was employed to obtain a sample size. Based on an observed food insecurity prevalence of 31.1% in Matiari, and using a simple asymptotic formula, a sample size of 224 villages was required to produce estimates at 5% precision and 95% level of confidence (Table 2). A listing of all villages (*N* = 585) in the district was generated by a previous intervention trial [30]. The present study used a random number generator to select villages from this list, and this was further stratified by village size (based on population percentiles, Figure 1). Using a 500-m Euclidean buffer radius from the village center, food outlets were surveyed for accessibility, availability of 15 food groups (fruits, vegetables, roots/tubers, legumes/pulses, nuts, eggs, milk, fish, meat/poultry, water, fruit juice, sugar-sweetened beverages, chips, sweets, and fast-food) and promotion. In the present study, “healthy” food was defined as nutrient-rich perishable food and beverage items that promote health, including fruits, vegetables, roots and tubers, legumes and nuts, eggs, milk, water, fish and seafood, and flesh and organ meat. “Unhealthy” food was defined as nutrient-poor (i.e., high in sugar, salt, and or fat) food and beverage items that may cause risk to health, including ultra-processed packaged foods and beverages such as sweetened soda or sweetened juice drinks, sugary foods such as chocolate bars, candies, cookies, cakes, and chips, and street and fast-food [31]. These definitions are consistent with World Health Organization’s classification of healthy and unhealthy diets [32]. All data were collected on mobile devices, with integrated GPS (Samsung Tablet A, SM-T285, using REDCap Mobile, Version 4.9.1 with built-in logic and range checks) from October 2020 to April 2021, which allowed the capture of latitude and longitude coordinates. 

### 2.4. Statistical Analysis

Descriptive statistics (means, standard deviations (SD), counts, and percentages) were used to describe food environment constructs (food accessibility, food availability, and food promotion) of food outlets. To test whether key constructs differed statistically by village size, a χ^2^ test or one-way ANOVA were conducted depending on categorical or continuous nature of variables, respectively. Type I error was set to 0.05 for all analyses. All data management and analyses were conducted in the software package SPSS, version 27 (IBM Corporation, Armonk, NY, United States).

## 3. Results

We sampled 224 villages (*n* = 54, small; *n* = 112, medium; *n* = 58, large) and a total of 1484 food outlets. See Table 3 for Characteristics. 

### 3.1. Accessibility

Of the total food outlets, the majority were mobile food vendors (37.7%) or corner stores (34.8%). Most food outlets operated all days of the week (67.9%), though often only during daytime hours (85.6%). Further, in large villages, most food outlets were under a 10-min walk (76.1%) from the nearest school, whereas in medium villages, food outlets were either directly outside of schools (41.1%) or under a 5-min walk (26.1%). In small and medium-sized villages, mobile food vendors were the predominant food outlet type, whereas in large villages, corner stores were more prominent. 

### 3.2. Availability

**Figure 2:**
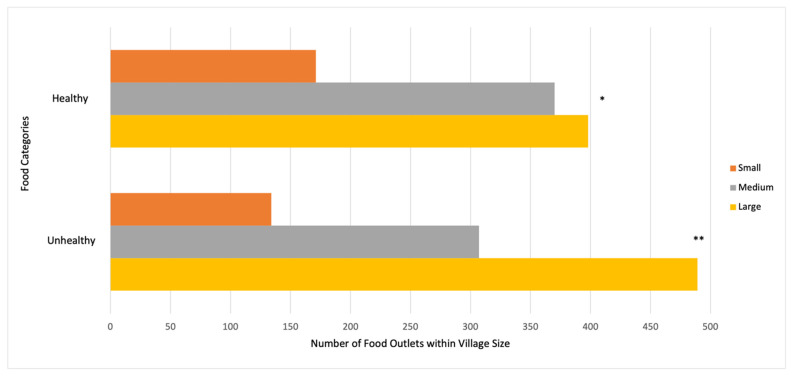
Availability of Healthy and Unhealthy Food by Total Number of Food Outlets in Villages. Healthy food consists of availability of fruits, vegetables, roots and tubers, legumes and nuts, eggs, milk, water, fish and seafood, and flesh and organ meat. Unhealthy food consists of ultra-processed food such as sweetened soda or sweetened juice drinks, sugary foods such as chocolates, candies, cookies, cakes, and chips, and street and fast foods. χ^2^ test * = *p* < 0.05; ** = *p* < 0.001.

**Figure 3:**
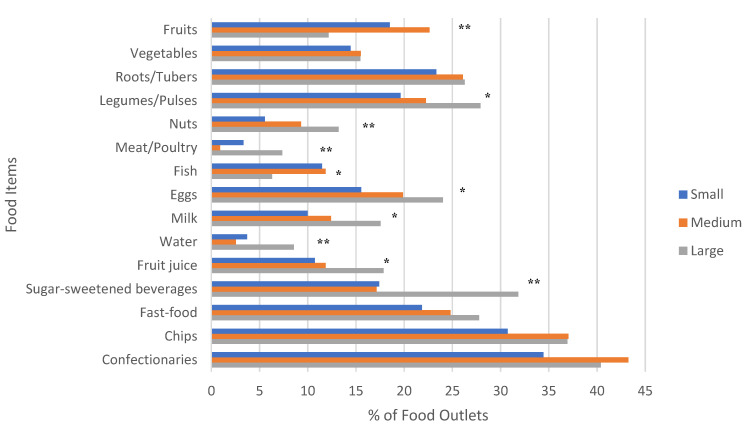
Proportion of Food Outlets with Available Food Items by Village Size. χ^2^ test, * = *p* < 0.01; ** = *p* < 0.001.

As illustrated in Figure 2, the number of food outlets selling healthy χ^2^ (2, *n* = 939) 7.79, *p* = 0.02 or unhealthy foods χ^2^ (2, *n* = 930) 62.90, *p* < 0.001 in small, medium, and large villages significantly differed. A one-way ANOVA revealed that the mean number of unhealthy food groups sold by food outlets by village size was significant (F(2, 1481) = 7.91, *p* < 0.001), with an increasing number of food groups available with increasing size of the village. Though a similar trend was observed, the mean number of healthy food groups did not significantly differ by village size (F(2, 1481) = 2.96, *p* < 0.052) (Table 3). The proportion of food outlets with available fruits, nuts, meat and poultry, water, and sugar-sweetened beverages significantly differed by village size (*p* < 0.001), as well as legumes/pulses, eggs, fish, milk and fruit juice (*p* < 0.01) (Figure 3). When examining food availability by food outlet type, corner stores sold a variety of healthy foods, especially vegetables, roots/tubers, legumes/pulses, nuts, eggs, and milk, but were also the primary source for unhealthy packaged foods (e.g., chips, sweets, sugar-sweetened beverages) (Table 4). Approximately 64.2% of corner stores were outside or under a 5-min walk from schools. Mobile food vendors sold mainly fruit and fast-food, while specialized vendors sold only fish (*n* = 113), sugar (*n* = 10), dates (*n* = 11), naan/chapati (*n* = 11), tea (*n* = 23), and pickles (*n* = 30) (data not shown).

The proportion of food outlets with a diversity of fruit offerings including vitamin A-rich fruits (e.g., mango, papaya, apricots), citrus fruits, bananas, apples, and other types (berries, guava) differed by village size (*p* < 0.001). In contrast, vitamin A-rich vegetables (e.g., pumpkin, peppers, sweet potato) (*p* = 0.943), and dark green leafy vegetables (*p* = 0.733), did not significantly differ by village size (Supplementary Table S1). Importantly, fruits and vegetables were most often sold by mobile vendors and corner stores (data not shown). The proportion of food outlets with ultra-processed and fast foods offerings, including ice cream (*p* = 0.042), chocolate cake (*p* = 0.004), laee (*p* = 0.018), kulfi (*p* < 0.001), cupcakes (*p* = 0.012), namak para (*p* = 0.003), nimko (*p* < 0.001), and pakoras (*p* = 0.018), significantly differed by village size (Supplementary Table S1). 

### 3.3. Promotion

For unhealthy packaged foods, we surveyed the types of brands available. For sugar-sweetened beverages, Pepsi and Mountain Dew were the most frequently available in approximately 15% of food outlets in all villages combined, but this differed by village size (*p* < 0.001 and *p* = 0.004, respectively) (Supplementary Table S2).

## 4. Discussion

To our knowledge, this is the first study to characterize retail food environments in a peri-urban district of Sindh, Pakistan. Though the intention was to collect data prior to the pandemic, our findings suggest that Matiari may have been insulated from COVID-19-related food supply chain disruptions, as a variety of healthy and unhealthy food items were available, and this was significantly associated with village size (Figure 2). However, the extent to which food environments were disrupted during COVID-19 cannot be concluded from our findings, and there is a lack of comparable data prior to the pandemic. Importantly, the variety of fruits, but not vegetables, was associated with village size. As illustrated in Table 3 and Figure 3, large villages had greater availability of most food items, except for fruits. This finding suggests that in large villages, where food value chains are presumably longer (as compared to small villages), fruits may have been affected by the global demand and supply shocks observed during COVID-19 [33,34]. Moreover, the availability and convenience of brand-name ultra-processed packaged foods and fast foods in all village sizes of our peri-urban district signals that modern value chains serving national and regional markets, particularly in urban areas, now complement traditional markets. Although we did not measure village market access, the authors recognize this is an important indicator to measure in future surveys to understand the food supply chain [35]. 

Our results are substantiated by country case studies conducted by World Food Programme on urban food systems during COVID-19, in eight cities in the Asia and Pacific regions (e.g., Peshawar, Pakistan; Kabul, Afghanistan; and Cox’s Bazar, Bangladesh) [36]. Their findings revealed two themes that are important considerations in understanding resilience of rural and peri-urban (the present study) food environments and ultimately the impact on food access and availability. First, urban areas were more susceptible to food supply chain disruptions, given their length and complexity, as compared to rural areas. Second, most cities showed high levels of absorptive capacity (low resilience). The combination of increased food prices and loss of income affected food affordability for the urban poor. Daily wage and informal sector workers were most affected, in part because they were not registered for social protection programmes [36]. 

As in other South Asian countries, the ‘hidden middle’ of Pakistan’s agrifood system, specifically the informal food sector and MSMEs, have revolutionized the wholesale, processing, and distribution of food [37,38] and proved to be critical to ensuring food security during COVID-19 [39]. In the present study, corner stores were a ‘one-stop shop’ for a variety of fresh produce and staple foods and were also the primary source for many ultra-processed packaged foods. This finding has implications for future policy initiatives, particularly as a large proportion of corner stores were located near schools in large villages. Moreover, our study revealed that small and medium villages were concentrated with mobile food vendors, which mainly sold fresh produce (e.g., fruits, vegetables, and roots/tubers) and fast-food. It is possible that during the pandemic, mobile food vendors internally migrated based on business prospects and potentially increased the number of food items for sale to accommodate consumers. There is also evidence to suggest that during periods of economic crisis, the informal sector inflates due to reduced formal employment opportunities and purchasing power, becoming a source of both income and food security [40]. For these reasons, governments and other food actors should consider informal food vendors as agents of change in food systems transformation and development initiatives.

In the case of Pakistan, 42% of the labour force is employed in agriculture, though food insecurity remains staggeringly high at 36.9% nationally [9,41]. During the pandemic, the Government of Pakistan introduced the Ehsaas Emergency Cash Program in April 2020 to address the economic burden placed on the poor and vulnerable, while sustaining basic dietary needs and marginally increasing health spending. At the consumer interface, the federal and provincial governments sought to keep food sales unrestricted during the lockdown and penalized hoarding and price gouging by retailers; this discouraged panic-buying of food [11]. However, anecdotal evidence suggests that widespread disruption and a series of localized lockdowns affected agricultural production and therefore downstream effects on the food environment. Empirical evidence from the Asian Development Bank suggests that the COVID-19 pandemic had negative impacts on farm households in the province of Sindh, Pakistan, including reduced demand for perishables and increased prices of farm inputs, particularly seeds, as well as locust invasion and crop damage during the kharif season (crops that are sown and harvested during the monsoon season, which lasts from about June to November) [42]. Furthermore, market research in Peshawar, Pakistan in December 2020–January 2021 by the Global Alliance for Improved Nutrition reported that food vendors stated their sales had decreased during the pandemic, and 40% of vendors used marketing strategies (additional discount, advertisement, sales on credit) to increase the number of customers or sales during this time [43]. 

With regards to the present study, various limitations exist, particularly related to survey methodology. First, as this study is cross-sectional in design, this precludes causal inference. Second, reliable and validated tools specific to Pakistan and our population of interest simply do not exist. Thus, local expert consultation and pretesting of developed tools were paramount to the success of this survey. Third, given that our study design was modified due to the pandemic and food environment data collection was conducted during the COVID-19 pandemic, we are unsure how this compares to food access and availability pre-pandemic, as well as rural, peri-urban, and urban differences. Finally, due to the modification in study design, this study was limited to a few indicators related to physical access of food and food promotion. There is recognition in the literature that economic and social access, for example one’s position in society (e.g., gender, class) and other forms of food marketing (e.g., social media) dramatically affects consumer behaviour and food choice, which further reinforces access and availability of food within the food environment (i.e., positive feedback loop) [44,45,46]. 

Future research should look to use mixed methods to generate a holistic narrative of food environments in Pakistan, by both expanding the number of quantitative indicators within the current survey tool and supplementing with qualitative research to deconstruct complex and dynamic interactions. Additionally, prospective studies should test the reliability and validity of the survey tool as a means to produce high-quality data, which will inform well-designed and targeted public health programs and policies. Repeating the survey would also allow for multiple data points (i.e., during crises and business as usual) to examine if and how the landscape of food and food retailers changed during the pandemic in Matiari and allow for reflection and response planning, including investments into real-time monitoring mechanisms, to strengthen food system resilience ahead of future shocks [47]. 

## 5. Conclusions

The global COVID-19 pandemic reaffirmed the need for transformation, by exposing the fragility of national, regional, and local food systems and the challenges faced in maintaining adequate food supplies, food purchasing, and protecting public health and nutrition in times of crisis [33]. Our findings revealed that mobile food vendors and corner stores were main food access points across small, medium, and large villages and were sources of both healthy and unhealthy foods during the height of the pandemic. As such, governments and food system actors should establish policies to promote the distribution of micronutrient-rich foods through the informal sector and MSMEs to build enabling environments. As well, linking these foods to social protection programs within retail food environments will help reach vulnerable segments of the population to ensure food security. Furthermore, our study established that brand-name, ultra-processed foods and beverages are existent in peri-urban food environments such as Matiari district, indicating the need for regulatory measures on the formulation, sale, and marketing of unhealthy foods, to ultimately curb the DBM and risk of non-communicable diseases. In conclusion, this study used a rigorous sampling strategy and culturally sensitive tool to explore and generate much needed scientific evidence on peri-urban food environments in a lower-middle income country context. It serves as an important model for neighbouring countries to undertake food environment research. 

## Figures and Tables

**Table 1:** Types of Food Outlets in Matiari, Pakistan. Adapted from [29].

Term	Definition
Food outlet	A food outlet includes all establishments where customers can buy food and drinks. These can include corner/convenience stores, fast-food outlets, wet markets, mobile/street/kiosk vendors, and restaurants.
Wet market	A wet market is a large outdoor, ‘open-air’ market, where individual vendors might sell fresh fruits and vegetables, as well as meat, fish, and grains.
Mobile food/street/kiosk vendor	A mobile food vendor, street vendor, or kiosk is usually run out of a motorized vehicles or non-motorized cart. These sorts of establishments are primarily engaged in preparing and serving meals and snacks for immediate consumption, like a specialty snack, such as pakoras, bun kebobs, or crisps; or serving non-alcoholic beverages, such as coffee, juices, or sodas. Consumption of the food or drink would usually happen at the vendor’s spot or nearby.
Small retailers	A corner or convenience store is a small store that sells some staple foods and a limited range of household goods.
Fast-food service	A fast-food outlet is usually a food retail shop with a limited menu that offers precooked or quickly prepared foods. In these shops, customers generally order or select items and pay before eating. Food and drink may be consumed on premises, taken out, or delivered to customers’ location.
Formal food service	Formal food service are outlets where people pay to sit and eat a meal that has been cooked on the premises and served to them (i.e., restaurants and cafes). Customers will usually order food while seated from a server and pay after eating. Sometimes restaurants may provide take away service.

**Table 2:** Sampling Frame.

Strata	Description	Population	Number of Villages	Number of People Per Village	% Food Insecure	Distribution of Villages (Per Strata)
1	Small villages (<25% of population)	3362	144	58	30%	55
2	Medium villages (25–75% of population)	60376	295	59–490	27%	113
3	Large villages (>75% of population)	281708	146	>490	29%	56
Total		345446	585			
Sample						224

**Table 3:** Food Outlet Accessibility and Availability by Village Size. Small (*n* = 54), Medium (*n* = 112), Large (*n* = 58). χ^2^ test for categorical variables; ANOVA for continuous variables.

Accessibility				
	Small	Medium	Large	*p*
**Food Outlets in Village Catchment *n* (%)**				**<0.001**
Mobile vendors (*n* = 560)	129 (47.8)	247 (45.1)	184 (27.6)	
Corner stores (*n* = 517)	74 (27.4)	203 (37.0)	240 (36.0)	
Specialized food (*n* = 244)	38 (14.1)	68 (12.4)	138 (20.7)	
Fast-food (*n* = 109)	16 (5.9)	21 (3.8)	72 (10.8)	
Restaurants (*n*= 49)	11 (4.1)	7 (1.3)	31 (4.6)	
Other (*n* = 5)	2 (0.7)	2 (0.4)	1 (0.2)	
Total (*n* = 1484)	270	548	666	
**Proximity to schools *n* (%)**				**<0.001**
Walking distance < 5 min	57 (21.1)	143 (26.1)	251 (37.7)	
Walking distance > 5 min	83 (30.7)	136 (24.8)	256 (38.4)	
Walking distance > 10 min	30 (11.1)	44 (8.0)	108 (16.2)	
Other (outside of school)	100 (37.0)	225 (41.1)	51(7.7)	
**Hours of operation *n* (%)**				**<0.001**
All hours	2 (0.7)	4 (0.7)	1 (0.2)	
Morning (7 a.m.–10 a.m.)	178 (65.9)	361 (65.9)	564 (84.7)	
Afternoon (12 p.m.–4 p.m.)	51(18.9)	75 (13.7)	42 (6.3)	
Evening (4 p.m.–close)	3 (1.1)	7 (1.3)	31(4.7)	
No fixed time	36 (13.3)	101 (18.4)	28 (4.2)	
**Days of operation *n* (%)**				**<0.001**
All days	152 (56.3)	308 (56.2)	548 (82.3)	
Weekdays	0	2 (0.4)	4 (0.6)	
Weekends	0	1 (0.2)	2 (0.3)	
Other ^a^	118 (43.7)	237 (43.2)	112 (16.8)	
**Availability**				
**Mean number of healthy food group items (SD)**	1.2 (1.4)	1.4 (1.5)	1.5 (1.8)	<0.052
**Mean number of unhealthy food group items (SD)**	1.2 (1.5)	1.3 (1.5)	1.6 (1.4)	**<0.001**
**Mean number of total food group items (SD)**	2.6 (2.4)	2.9 (2.6)	3.2 (2.8)	**<0.006**

^a^ Other responses for days of operation included: inconsistent days, alternate days, seasonal. Bolded *p* values indicate significance.

**Table 4:** Food Availability by Food Outlet Type.

	Type of Food Outlet				
Type of Food *n* (%)	Mobile Vendors (*n* = 560)	Corner Stores (*n* = 517)	Specialized Food (*n* = 244)	Fast-Food (*n* = 109)	Restaurants (*n* = 49)
Fruits	197 (35.2)	51 (9.9)	2 (0.8)	3 (2.8)	1 (2.0)
Vegetables	86 (15.4)	106 (20.5)	3 (1.2)	3 (2.8)	29 (59.8)
Roots/Tubers	85 (15.2)	261 (50.5)	3 (1.2)	6 (5.5)	26 (53.1)
Legumes/Pulses	4 (0.7)	316 (61.1)	0	2 (1.8)	39 (79.6)
Nuts	6 (1.1)	147 (28.4)	0	1 (0.9)	0
Meat/Poultry	1 (0.2)	0	38 (15.6)	5 (4.6)	19 (38.8)
Fish	22 (3.9)	0	113 (46.3)	1 (0.9)	2 (4.1)
Eggs	1 (0.2)	282 (54.5)	1 (0.4)	4 (3.7)	23 (46.9)
Milk	0	182 (35.2)	11 (4.5)	2 (1.8)	17 (34.7)
Water	1 (0.2)	63 (12.2)	2 (0.8)	5 (4.6)	10 (20.4)
Fruit juice	0	187 (36.2)	5 (2.0)	9 (8.3)	12 (24.5)
Sugar-sweetened beverages	0	238 (46.0)	75 (30.7)	12 (11.0)	28 (57.1)
Fast-food	140 (25.0)	122 (23.6)	2 (0.8)	107 (98.2)	8 (16.3)
Chips	17 (3.0)	493 (95.4)	3 (1.2)	4 (3.7)	13 (26.5)
Confectionaries	57 (10.2)	499 (96.5)	7 (2.9)	20 (18.3)	14 (28.6)
**Proximity to schools *n* (%)**					
Outside of school	217 (38.8)	106 (20.5)	42 (17.2)	5 (4.6)	2 (4.1)
Walking distance < 5 min	102 (18.2)	226 (43.7)	71 (29.1)	39 (35.8)	13 (26.5)
Walking distance > 5 min	193 (34.4)	125 (24.2)	86 (35.2)	43 (39.4)	24 (49.0)
Walking distance > 10 min	46 (8.2)	59 (11.4)	45 (18.4)	22 (20.2)	10 (20.4)

## Data Availability

Not applicable.

## Supplementary Materials

The following supporting information can be downloaded at: https://www.mdpi.com/article/10.3390/ijerph19148614/s1, Table S1: Diversity of Foods Available by Village Size; Table S2: Variety and Brands of Unhealthy Foods Available by Village Size.
