# Supplementary material for: Characterizing Retail Food Environments in Peri-Urban Pakistan during the COVID-19 Pandemic
Source: Int J Environ Res Public Health. 2022 Jul 15;19(14):8614. doi: 10.3390/ijerph19148614 (PMC9324779; doi:10.3390/ijerph19148614)
Supplement: Supplementary file 1 [file ijerph-19-08614-s001.zip › ijerph-1758552-supplementary.pdf]

Supplementary File

Table S1. Diversity of Foods Available by Village Size
χ^2 *test* for differences in village size

**Food outlets in village catchment n (%)**	**Small**	**Medium**	**Large**	***p***
**Fruits**				
Vitamin-A rich fruits	27 (10.0)	62 (11.3)	35 (5.25)	**<0.001**
Citrus fruits	44 (16.3)	116 (21.2)	64 (9.6)	**<0.001**
Bananas	46 (17.0)	101 (18.4)	65 (9.8)	**<0.001**
Apples	37 (13.7)	93 (17.0)	58 (8.7)	**<0.001**
Other (berries, guava)	37 (13.7)	97 (17.7)	48 (7.2)	**<0.001**
**Vegetables**				
Dark leafy green vegetables	31 (11.5)	65 (11.9)	87 (13.1)	0.733
Vitamin A-rich vegetables	33 (12.2)	63 (11.5)	80 (12.0)	0.943
**Fast-foods**				
Namak para	4 (1.5)	3 (0.5)	21 (3.2)	**0.003**
Patties	5 (1.9)	10 (1.8)	21 (3.2)	0.259
Biryani	6 (2.2)	15 (2.7)	29 (4.4)	0.153
Chola chat	8 (3.0)	21 (3.8)	26 (3.9)	0.773
Khecha baja	8 (3.0)	15 (2.7)	13 (2.0)	0.553
Samosas	15 (5.6)	35 (6.4)	60 (9.0)	0.097
Pakora	19 (7.0)	25 (4.6)	58 (8.7)	**0.018**
Nimko	19 (7.0)	58 (10.6)	30 (4.5)	**<0.001**
**Confectionaries**				
Balu shahi	2 (0.7)	10 (1.8)	17 (2.6)	0.186
Jalebi	3 (1.1)	5 (0.9)	14 (2.1)	0.280
Puff pastry	5 (1.9)	17 (3.1)	15 (2.3)	0.484
Boondi	7 (2.6)	7 (1.3)	17 (2.6)	0.247
Lacho	10 (3.7)	29 (5.3)	35 (5.3)	0.563
Ice Cream	12 (4.4)	13 (2.4)	11 (1.7)	**0.042**
Gulab Jamun	14 (5.2)	37 (6.8)	52 (7.8)	0.351
Chocolate cake	14 (5.2)	27 (4.9)	63 (9.5)	**0.004**
Barfi	26 (9.6)	79 (14.4)	101 (15.2)	0.077
Sohan halwa	26 (9.6)	66 (12.0)	86 (12.9)	0.375
Sponge cake	33 (12.2)	76 (13.9)	90 (13.5)	0.805
Kulfi	34 (12.6)	48 (8.8)	23 (3.5)	**<0.001**
Laee	39 (14.4)	125 (22.8)	140 (21.0)	**0.018**
Cupcake	55 (20.4)	132 (24.1)	194 (29.1)	**0.012**

Table S2. Variety and Brands of Unhealthy Foods Available by Village Size
χ^2 *test* for differences in village size

**Food outlets in village catchment n (%)**	**Small**	**Medium**	**Large**	***p***
**Sugar-sweetened Beverages**				
Nauras	1 (0.4)	3 (0.5)	11 (1.7)	0.081
Lassi	2 (0.7)	1 (0.2)	6 (0.9)	0.263
Pakola Soda	6 (2.2)	12 (2.2)	60 (9.0)	**<0.001**
Rooh Afza	8 (3.0)	12 (2.2)	42 (6.3)	**<0.001**
Jam-e-Sheeri (Qarshi)	13 (4.8)	28 (5.1)	70 (10.5)	**<0.001**
Al Tunsa	14 (5.2)	32 (5.8)	66 (9.9)	**0.008**
Tang	19 (7.0)	34 (6.2)	59 (8.9)	0.206
Sting	21 (7.8)	35 (6.4)	85 (12.8)	**<0.001**
Marinda	29 (10.7)	64 (11.7)	121 (18.2)	**<0.001**
7up	29 (10.7)	66 (12.0)	121 (18.2)	**0.002**
Sting	21 (7.8)	35 (6.4)	85 (12.8)	**<0.001**
Pepsi	31 (11.5)	66 (12.0)	128 (19.2)	**<0.001**
Mountain Dew	33 (12.2)	67 (12.2)	123 (18.5)	**0.004**
**Fruit Juice**				
Nestle	7 (2.6)	15 (2.7)	50 (7.5)	**<0.001**
Pakola Juice	18 (6.7)	41 (7.5)	86 (12.9)	**<0.001**
Slice	20 (7.4)	26 (4.7)	76 (11.4)	**<0.001**
Popular Polly	29 (10.7)	56 (10.2)	114 (17.1)	**<0.001**
**Sweets**				
Jubilee	10 (3.7)	21 (3.8)	34 (5.1)	0.467
Dairy milk	12 (4.4)	23 (4.2)	57 (8.6)	**0.003**
Cadbury eclairs	59 (21.9)	136 (24.8)	174 (26.1)	0.391
